# Phenotypic and Functional Characterization of Human Mammary Stem/Progenitor Cells in Long Term Culture

**DOI:** 10.1371/journal.pone.0005329

**Published:** 2009-04-24

**Authors:** Devaveena Dey, Meera Saxena, Anurag N. Paranjape, Visalakshi Krishnan, Rajashekhar Giraddi, M. Vijaya Kumar, Geetashree Mukherjee, Annapoorni Rangarajan

**Affiliations:** 1 Department of Molecular Reproduction, Development and Genetics, Indian Institute of Science, Bangalore, India; 2 Department of Pathology, Kidwai Memorial Institute of Oncology, Bangalore, India; McMaster University, Canada

## Abstract

**Background:**

Cancer stem cells exhibit close resemblance to normal stem cells in phenotype as well as function. Hence, studying normal stem cell behavior is important in understanding cancer pathogenesis. It has recently been shown that human breast stem cells can be enriched in suspension cultures as mammospheres. However, little is known about the behavior of these cells in long-term cultures. Since extensive self-renewal potential is the hallmark of stem cells, we undertook a detailed phenotypic and functional characterization of human mammospheres over long-term passages.

**Methodology:**

Single cell suspensions derived from human breast ‘organoids’ were seeded in ultra low attachment plates in serum free media. Resulting primary mammospheres after a week (termed T1 mammospheres) were subjected to passaging every 7th day leading to the generation of T2, T3, and T4 mammospheres.

**Principal Findings:**

We show that primary mammospheres contain a distinct side-population (SP) that displays a CD24^low^/CD44^low^ phenotype, but fails to generate mammospheres. Instead, the mammosphere-initiating potential rests within the CD44^high^/CD24^low^ cells, in keeping with the phenotype of breast cancer-initiating cells. In serial sphere formation assays we find that even though primary (T1) mammospheres show telomerase activity and fourth passage T4 spheres contain label-retaining cells, they fail to initiate new mammospheres beyond T5. With increasing passages, mammospheres showed an increase in smaller sized spheres, reduction in proliferation potential and sphere forming efficiency, and increased differentiation towards the myoepithelial lineage. Significantly, staining for senescence-associated β-galactosidase activity revealed a dramatic increase in the number of senescent cells with passage, which might in part explain the inability to continuously generate mammospheres in culture.

**Conclusions:**

Thus, the self-renewal potential of human breast stem cells is exhausted within five in vitro passages of mammospheres, suggesting the need for further improvisation in culture conditions for their long-term maintenance.

## Introduction

Stem cells are found in many adult tissues and play a critical role in tissue homeostasis throughout life. Although few in an organism, these cells can repopulate an entire tissue when required, such as during injury or disease, by virtue of their long term self-renewal potential and the ability to generate heterogeneous progeny [1]. However, the long life span coupled with extensive proliferation potential of several adult stem cells also makes them attractive candidates for accumulating multiple mutations. The recent identification of a rare population of cancer stem cells within several cancers [2]–[6], with both phenotypic and functional resemblances to normal stem cells, further supports the notion that normal stem cells may be the cellular origin of cancer. Therefore understanding stem cell behavior is fundamental not only for our understanding of normal development but also carcinogenesis.

Both normal and cancer stem cells have been identified in the mammary gland [7]–[10]. The adult mammary gland, organized as a ‘mammary tree’, has a lobulo-alveolar structure, composed of luminal cells that lie along the interior surfaces of ducts and alveoli, and myoepithelial cells underlying the basement membrane of the ductal-alveolar network [11]. Functional evidence for the presence of mammary epithelial stem cells was first demonstrated in rodents by transplantation studies that showed generation of functional mammary tree by transplantation of mammary outgrowths into cleared fat pads of syngeneic mice [12]. More recently, mammary stem cells have been identified based on expression of a combination of cell surface markers such as Sca-1, CD24 and CD49f. Indeed a single murine mammary stem cell was shown to generate a complete, functional mammary gland in vivo [13], [14], thereby demonstrating their long term self-renewal potential as well as their ability to differentiate into all the cell types that form a normal functional mammary gland.

Evidence for the existence of stem cells in human mammary gland came from studies of X chromosome inactivation. Adjacent patches of the epithelium had inactivation of the same X chromosome, indicating that these cells arose from a single primitive cell [15], [16]. Further evidence came from the observation that the same genetic lesion could be detected in an entire duct or lobule of a histologically normal mammary epithelium [17]. It was also demonstrated that luminal and myoepithelial cells from the same region of the breast show similar patterns of loss of heterozygosity, indicating a common progenitor [18]. Even though some putative markers such as p21, Musashi 1, CD49f, Cytokeratin 19 (CK19), Bmi-1 and ESA^+^Muc1^−^ [10] have been suggested for human breast stem cells, no definitive markers have yet been identified, thereby limiting their isolation and characterization.

The inability to maintain adult stem cells in an undifferentiated state in vitro has further marred their characterization. Recently, enrichment of human breast stem/progenitor cells as non-adherent mammospheres in serum-free media was demonstrated [19], based on the strategy adopted for neuronal stem cell culture as neurospheres [20]. Primary mammosphere-derived cells can initiate secondary mammospheres, as well as differentiate to give rise to various breast lineages, thereby exhibiting two fundamental properties of stem cells – self-renewal and multilineage differentiation. Additionally, both murine and human mammospheres have the potential to generate ductal-alveolar outgrowths in vivo, further demonstrating the existence of stem cells within these units [21], [22]. Thus, the mammosphere system offers an in vitro model to study mammary stem cell biology. Indeed, the involvement of Notch and Hedgehog signaling pathways in regulating mammary stem cell self-renewal has been demonstrated using the mammosphere system [22], [23]. However, little is known about the behavior of breast stem cells within mammospheres in long-term cultures.

In order to better comprehend the mammosphere system for its potential use in long-term maintenance of stem cells in culture, we have undertaken a thorough phenotypic and functional characterization of mammospheres. We show that the side population and sphere forming potential resides within two distinct subpopulations of CD24/44 dual stained mammospheres. In serial sphere formation assays, we find variations in size, number, proliferation, and differentiation status of mammospheres over passages. Importantly, we fail to detect generation of new mammospheres beyond the fifth passage despite the presence of live, proliferating and label retaining cells. Staining for senescence-associated β-galactosidase activity revealed a dramatic increase in the number of senescent cells with passage, which might in part explain the inability of these cells to continuously generate mammospheres in culture.

## Materials and Methods

### Collection and processing of breast tissue

Normal breast tissue was obtained from Kidwai Memorial Institute of Oncology (KMIO) Bangalore, as per the Institutional Review Board, in compliance with the ethical guidelines of KMIO and the Indian Institute of Science. Patient consent was obtained in a written form prior to surgery, as per the protocol approved by the IRB of KMIO. Tissue was collected 6 cm away from or diagonally opposite to tumor site by pathologists at KMIO from mastectomy cases. H & E staining on the tissues confirmed their normal state. Tissues were collected in DMEM (minus phenol red) containing penicillin, streptomycin, gentamycin and fungizone and dissociated mechanically and enzymatically using 1 mg/ml Collagenase (Sigma Aldrich) and 100 U/ml Hyaluronidase (Calbiochem) at 37°C for 16–18 hours with rotation. Breast organoids were separated by differential centrifugation and seeded in 50-mm low attachment plate in serum free DMEM-F12 media containing 10 ng/ml hEGF, 1 µg/ml hydrocortisone, 10 µg/ml insulin, 20 ng/ml bFGF, 4 ng/ml heparin (Sigma Aldrich), B27 (Invitrogen) as described earlier [19] supplemented with antibiotics.

### Culture of mammospheres

The organoids were enzymatically dissociated into single cells after 6–8 hrs in culture and typically 2.5×10^5^ cells seeded per well in 6-well ultra low attachment plates (Corning). Mammospheres formed after 7 days were collected by centrifugation at 1000 rpm. For serial passaging, total number of mammospheres obtained at each passage was counted microscopically under a manually prepared ‘quadrant grid’, enzymatically dissociated into largely single cells, and seeded again in low attachment plates after live cell count. Sphere formation efficiency at each passage was calculated by dividing the total number of spheres formed by the total number of live cells seeded multiplied by hundred.

### Immunocytochemistry and microscopy

Staining of intact spheres was done, both in suspension and by fixation onto coated slides. Fixation was done with 1∶1 −20°C prechilled Methanol: Acetone for 10 mins. at room temperature (RT). Prior to permeabilsation, a short trypsin treatment was done to the fixed spheres for 3–5 mins., only for intracellular antigens. No such treatment was done for cell surface antigen like E-Cadherin. Permeabilisation was done in conjunction with blocking in 0.2% Fish Skin Gelatin using 0.5% Triton ×100 for 1 hr. This was followed by incubation with primary antibody for 2 hrs at RT. Primary antibodies were used at dilutions as specified by the manufacturer. Secondary antibody directly conjugated to a suitable flurophore was added and incubated for 45 min at room temperature in dark. Propidium Iodide (PI) was added as counter stain at a concentration of 0.5 ug/ml with the last PBS wash, incubated for 5 mins. at room temperature before mounting. DABCO was used as an antifade agent in the mounting media. Edges were sealed & the slides viewed using Zeiss 510 Meta confocal laser scanning microscope and analyzed using LSM Image Browser. For single cell staining, spheres were first trypsinised, fixed on slides, stained as above and viewed under fluorescence microscope (Leica). Antibodies against E Cadherin (BD Biosciences); ESA, Cytokeratins 14 and 18 (Sigma Aldrich); Cytokeratin 19 (Calbiochem) were used.

### Confocal Imaging & Image Analysis

A Zeiss 510 Meta confocal laser scanning microscope was used to view the immunoflourescence staining. The 488, 543 or 660 nm laser lines were used for excitation of the fluorophores, while emissions were collected by specific band pass filters. Since the spheres are 3 dimensional specimens, optical sectioning was done along the XZ-plane to get a Z stack of the specimen.

### SP analysis

Standardisation of the protocol for SP was done in HeLa cells and C57/black BL6 mouse bone marrow cells. After trypsinisation of cells, 5×10^5^ cells was given a PBS wash and incubated with 1 ml of DMEM-F12 media and incubated at 37°C for 15 min. This was followed by addition of Hoechst 33342 at a final concentration of 2.5 µg/ml. To block Hoechst efflux, either Verapamil was added at a final concentration of 50 µM or Cyclosporin A was added at a concentration of 20 µM. The tubes were incubated in the dark at 37°C and 5% CO_2_ for 120 mins. with a constant and slow agitation. This was followed by two washes with PBS. For Rhodamine 123 staining, the dye was added upto a final conc. of 0.5 µg/ml and incubated at 37°C for 45 mins. This was followed by incubation in Rhodamine free medium for 30 mins. Cells were given a wash with cold PBS after staining and were analysed on the LSR-II machine (Becton Dickinson) . Hoechst 33342 was excited with a 355 nm UV laser. Emissions were collected in the blue and red channels using the 440/40 BP and 675 LP filters respectively. Immediately before analysis, Propidium Iodide (PI) was added at a final concentration of 2 µg/ml.

### CD44 and CD24 expression analysis on mammosphere cells

Mammosphere cells were incubated with anti CD24-FITC and anti CD44-PE antibodies (Becton Dickinson) in dark, on ice, for 45 mins, washed twice with cold PBS, resuspended in complete media and kept on ice till subsequent analysis on MoFlo (Dako). 50,000 cells from each quadrant were sorted in 1 ml complete media and seeded in 24 well Ultra low attachment plate (Corning). For dual staining of SP and CD markers, staining for SP was done at RT followed by staining for CD24 and 44 on ice as described above.

### Proliferation assay

BrdU was added immediately after seeding cells to a final concentration of 10 µM. Cells were incubated with BrdU for a week at 37°C & 5% CO_2_ during which they formed spheres. These spheres were then dissociated into single cells and stained with Anti-BrdU antibody. Fixation was done using 70% ethanol. HCl treatment was done to remove the histones. Secondary antibody used was conjugated with FITC. The other steps of staining were the same as described earlier. PI was used as counterstain. Staining for BrdU was done for mammospheres at every passage. For each staining experiment, a negative control was included, in which primary antibody had not been added. Imaging of intact spheres stained for BrdU was done on the confocal laser scanning microscope & optical sectioning was done as described above to analyse the staining profile in all the layers of a mammosphere. Imaging of stained single cells was done in a regular fluorescence microscope (Leica). In order to quantify the proliferation status of cells within mammospheres of each passage, 10 random fields were captured during imaging. Total cell count and total positive cell count was taken over the random fields post imaging. A grand total of all the fields was obtained by adding up numbers from the random fields. This was done for 3 independent tissues at each passage and a graph was plotted subsequently to represent the proliferation status of mammosphere derived cells through multiple passages.

### Label Retention Assay

BrdU (10 µM) was added to 2.5×10^5^ cells/ well of a 6-well ultra low attachment plate. At each passage (one week) a single well was harvested for BrdU staining, while the other wells were enzymatically dissociated and subjected to serial mammosphere assay without further addition of BrdU. Intact spheres from all the passages were subjected to BrdU staining and viewed by confocal microscopy.

### Senescence Assay for β - Galactosidase

Single cells obtained by enzymatic digestion of the spheres of each passage were given a wash with ice cold PBS followed by fixation with 0.2% glutaraldehyde at RT for 5 minutes. Post fixation, the cells were resuspended in buffer (pH 6.0) containing 1 mg/ml X-Gal and incubated in the dark at room temperature for 12 to 14 hours. The X-Gal was quenched by washing with ice cold PBS. The cells were viewed and counted under a regular phase contrast microscope.

## Results

### Mammospheres reveal cellular heterogeneity

**Figure 1:**
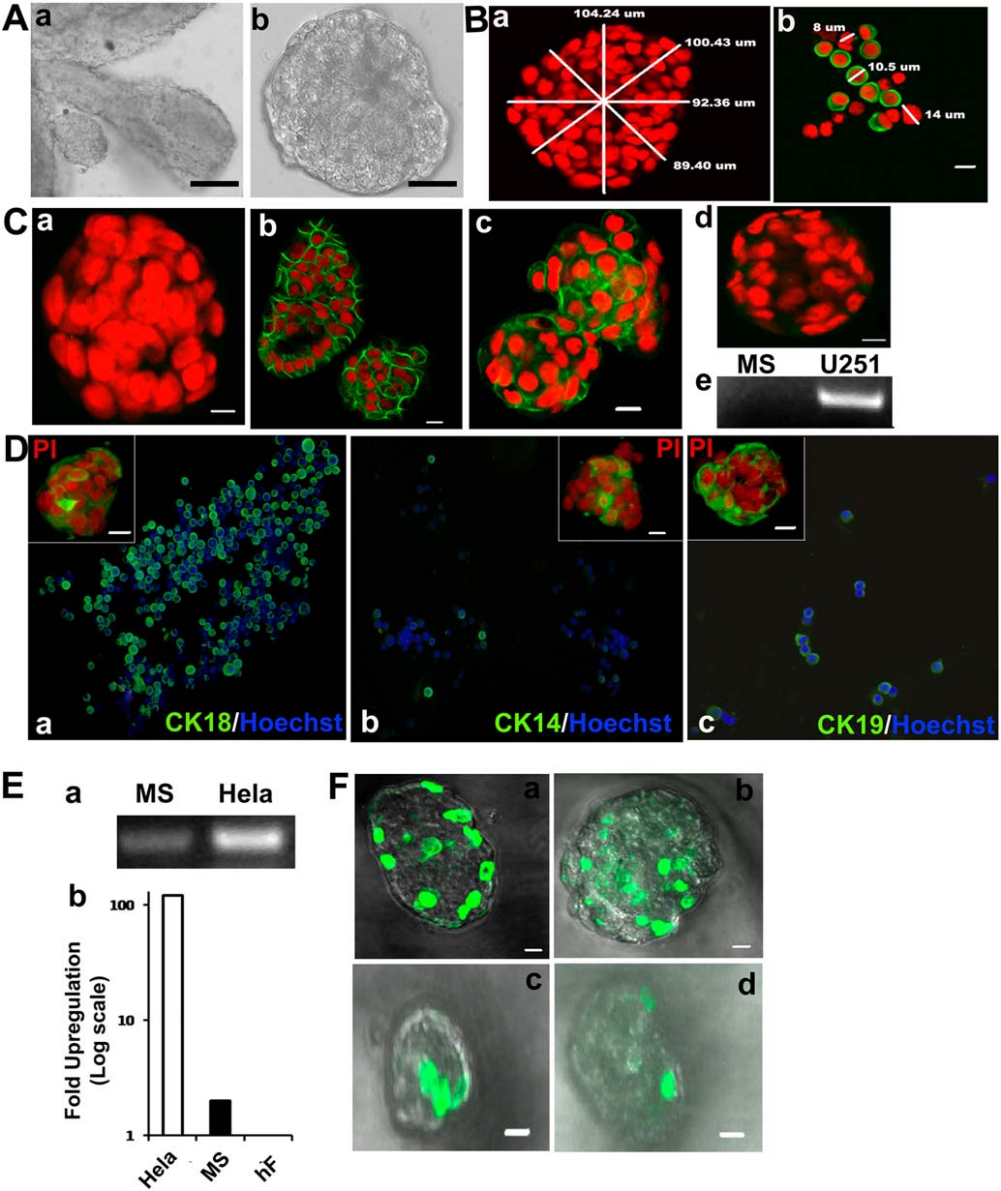
Phenotypic characterization of mammospheres. A: Representative phase contrast photomicrographs of organoids derived from primary human breast tissue; ×10 objective (a) and a primary mammosphere formed in suspension after 7 days; ×10 objective (b). B: Measurement of mammosphere size; ×10 objective (a) and cell size comprising the sphere (b) using LSM Image Browser from the Carl Zeiss website. C: Immunostaining of intact mammospheres viewed using a Zeiss 510 Meta confocal laser scanning microscope, and optical sectioning done along the XZ-plane to get a Z stack of the specimen. Photomicrographs represent negative control (minus primary antibody) (a), positive immunostaining for E-Cadherin (b) and Epithelial Specific Antigen (ESA) (c), but not for CD34 (d). RT-PCR analysis shows lack of nestin transcripts in mammospheres compared to U251 glioma cell line used as positive control (e). D: Photomicrographs represent immunostaining of single cells derived from primary mammospheres showing positivity for CK18 (a) CK14 (b) and CK19 (c). Insets show confocal images of intact spheres stained for the corresponding antigen. Hoechst 33342 and propidium iodide (PI) were used as nuclear counter stains for single cell and intact spheres, respectively (blue, Hoechst 33342; green, FITC; red, PI; scale bar = 20 µm). E: Gel picture shows hTERT expression in primary mammospheres (MS) by RT-PCR using HeLa as a positive control (a). Bar graph represents quantitative real time RT-PCR for hTERT expression in mammospheres compared to HeLa using primary human diploid fibroblasts (hF) as negative control (b). F: Mammospheres show few label retaining cells in T4 spheres. (a–d) represent photomicrographs of mammospheres immunostained for BrdU at each passage. (green, FITC signal; scale bar = 20 µm).

Normal breast tissue was subjected to mechanical disruption followed by enzymatic treatment that resulted in the release of breast ‘organoids’ (Fig. 1Aa). Further enzymatic digestion of the organoids yielded single cells that were seeded in low-attachment plates in media devoid of serum (see Materials and Methods). Floating spheroids, termed mammospheres, were observed within 5–7 days (Fig. 1Ab) as described previously [19]. Cells obtained by the enzymatic dissociation of mammospheres at each passage were further seeded under similar culture conditions for serial mammosphere assays. We have termed the enzymatic dissociation of organoids leading to the generation of primary mammospheres as ‘T1’, such that the subsequent passages of mammospheres were termed as T2, T3 etc. Accordingly, the spheres obtained after the first trypsinisation were termed as ‘T1’ spheres and so on. The time interval between each passaging was one week.

In order to understand the cellular architecture of mammospheres, we utilized confocal laser scanning microscopy to view and analyze cells in each plane through the entire sphere. Optical sections taken through propidium iodide (PI) stained spheres revealed that mammospheres are compact cellular structures (Fig. 1Ba). The mammospheres obtained after 7 days ranged in size from 40–110 µm in diameter, with the majority of spheres having a diameter of 75 µm. For a sphere of 110 µm diameter, the number of cells varied between 200 and 250, while a sphere of 40 µm contained ∼25 cells. The size of individual cells comprising the mammospheres varied from 9 to 15 µm (Fig. 1Bb), with ∼70% of the cells having a diameter between 12 and 15 µm. On analyzing the size of mammospheres through subsequent passages, we found that the size of the largest and smallest spheres in each passage remained consistent; however, the relative proportion of smaller spheres increased in number with passage (data not shown).

Further analysis of the cellular composition of mammospheres by immunocytochemistry revealed that when compared with the negative control (Fig. 1Ca), cells within the sphere stained positive for epithelial markers, (E)-Cadherin and ESA (Fig. 1Cb and c), while being negative for haematopoeitic and neuronal stem cell markers, CD34 and nestin (Fig. 1Cd and e). This indicates that the mammospheres are epithelial in nature and are not derived from haematopoeitic or neuronal stem cells. Staining for cytokeratin 14 and 18 (CK14 and 18) , which mark the myoepithelial and luminal cells, respectively, revealed positivity for both within primary mammospheres, albeit at varying proportions. While 70% cells were positive for CK18 (Fig. 1Da), 7% cells were positive for CK14 (Fig. 1Db). Additionally, staining for CK19, which marks early progenitors and luminal epithelial cells [24] revealed 30% of cells staining for CK 19 (Fig. 1Dc). Staining of intact spheres followed by Z-stack analysis by confocal microscopy revealed that these cytokeratins were uniformly distributed within the spheres with no particular preference for any cell type within the core or the periphery of the sphere (Supp Fig. S1A and B). Thus, primary mammospheres are composed of a heterogeneous population of cells containing both differentiated and undifferentiated cells.

### Mammospheres exhibit telomerase activity

While most somatic cells are devoid of detectable telomerase activity, some adult stem cells exhibit telomerase activity [25]. In order to test if the mammospheres have telomerase activity we carried out multiple assays in primary mammospheres. The spheres were found to be positive for telomerase expression as detected by RT-PCR analysis for the catalytic subunit of telomerase, hTERT (Fig. 1Ea). In addition, TRAP assay further revealed telomerase activity in primary and secondary mammospheres (Fig. 1Eb and Supp Fig. S1C and 1D). Unlike an earlier study that failed to detect telomerase activity by TRAP assay in primary breast tissue [26] we detected telomerase activity in primary mammospheres, indicating that mammospheres are enriched in stem cells.

### Mammospheres contain label-retaining cells

Adult stem cells have been identified in several systems by label retention assays wherein the slow dividing property of stem cells causes them to retain labels such as BrdU or tritiated thymidine, while the fast proliferating progenitors dilute them out with time [27]. In order to find if mammospheres contain such label retaining cells (LRCs), we pulsed mammospheres with BrdU for seven days and thereafter chased the label for a month with periodic passaging every 7^th^ day (Fig. 1F). At the end of first week, T1 mammospheres showed the presence of 5–10 BrdU positive cells per sphere (Fig. 1Fa). After one month of chase, 68% of the T4 spheres retained only 1–2 BrdU positive cells located mostly at the periphery of the spheres (Fig. 1Fd), while the rest contained none. Thus, mammospheres contain few label retaining cells at the end of one month that may represent putative stem cells.

### Mammospheres harbor Hoechst and Rhodamine effluxing cells

Many stem cells are endowed with the ability to efflux certain lipophilic drugs due to their cell surface expression of ABC family of membrane transporter proteins [28]. This property has been widely exploited to identify and isolate stem cells from several tissues, such as blood, brain, liver, muscle and others using Hoechst 33342 [29], [30]. The dye effluxing cells appear as a low fluorescing population, termed as the ‘Side Population’ (SP) [31]. In the presence of chemical inhibitors of the membrane transporters, like verapamil or cyclosporin A, this side population disappears. Another small molecule which has been reported to be effluxed by stem cells is Rhodamine123 [32] such that in a FACS histogram stem cells are marked by the Rhodamine-low (Rho^low^) population. Both SP and Rho^low^ fraction encompassing stem cells have been reported for multiple normal tissues [31]–[34].

**Figure 2:**
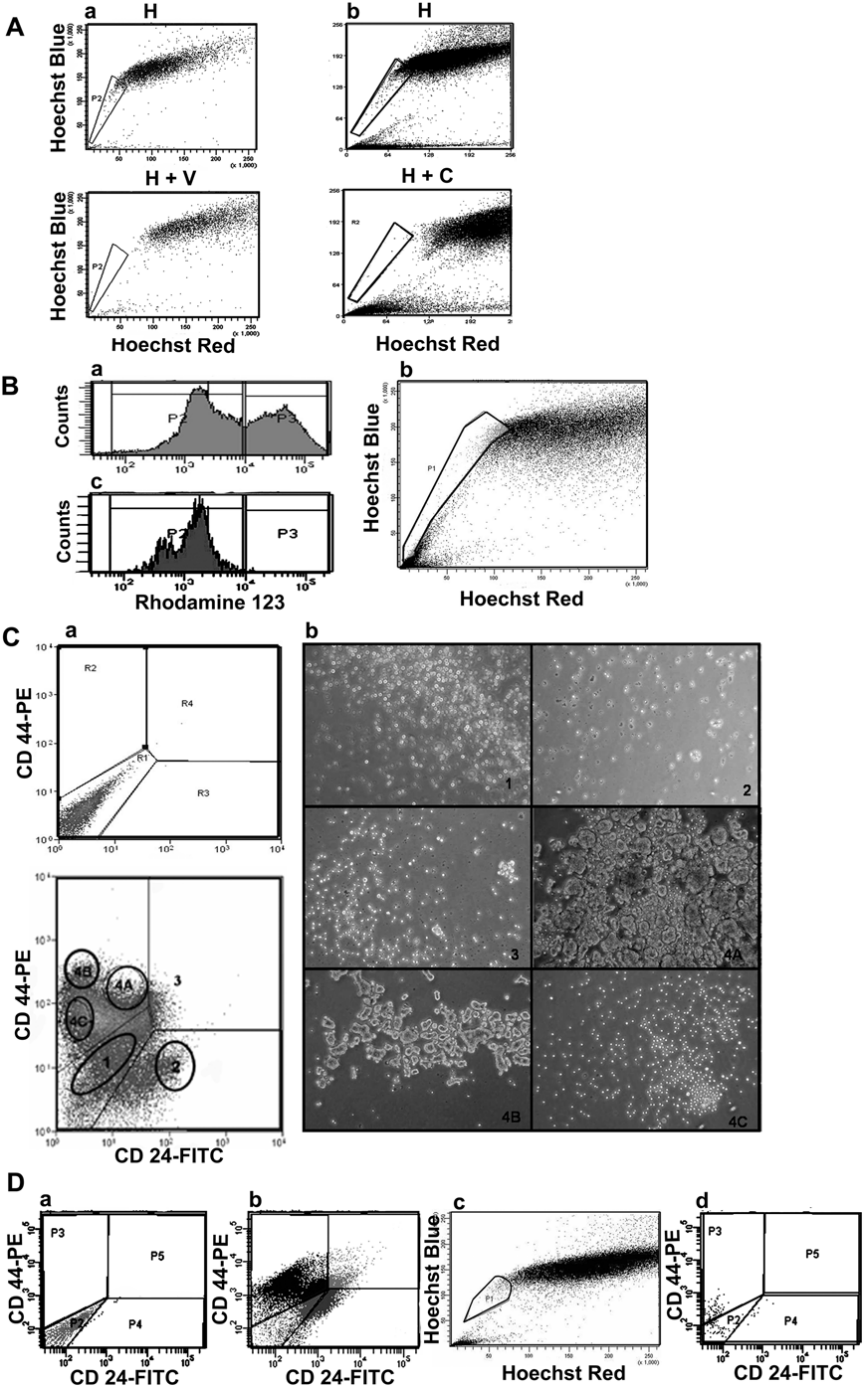
Analysis of efflux property and surface marker profile of primary mammospheres. A: Side Population (SP) analysis of primary mammosphere-derived cells from two individual samples (a and b) reveal distinct SP. The top panels represent cells treated with Hoechst 33342 only (H), while the bottom panels represent cells treated with ABC transporter inhibitors verapamil (H+V) or cyclosporine (H+C). B: FACS profile of primary mammosphere-derived cells treated with Rhodamine 123 (a). In a simultaneous treatment of cells with Hoechst and Rhodamine, the SP (b) gated onto the Rhodamine profile (c) revealed that SP falls in the Rho^low^ region. C: FACS profile of mammosphere-derived cells immunostained with anti CD24-FITC and anti CD44-PE antibodies (bottom) compared to unstained cells (top) (a). When 50,000 cells from different fractions were sorted and seeded in a 24-well ultra low attachment plate to assess sphere-forming ability (b), only the CD24^low^CD44^high^ fraction (4A and 4B) generated mammospheres. D: Mammosphere-derived cells, when simultaneously stained for CD24/44 and SP, revealed that SP falls in the CD24^low^CD44^low^ fraction. Dot plots represent unstained (a), CD24/44 profile (b), SP profile (c) and SP cells gated on to the CD24/44 profile (d).

When primary mammosphere-derived cells were subjected to SP analysis we observed a distinct side population comprising 0.5%–1% of the mammosphere cells, which disappeared on treatment with verapamil or cyclosporin A (Fig. 2A). A previous report had documented the presence of 30% SP cells within primary mammospheres [19]. However, in this study instead of a distinct population, a large fraction of cells, including Hoechst^low^ and Hoechst^high^ cells, disappeared in the presence of verapamil. We find that this experiment fluctuates with slight variations in concentration and time of incubation with Hoechst, pH and temperature. Accordingly, we did our experiments in parallel with mouse bone marrow samples where we constantly found a pattern of SP which is well established in the literature (Supp Fig. S2A and B) [31]. Similar to bone marrow, we find a tight SP population in the primary mammospheres. Thus, our experiments reveal that mammospheres contain a small fraction of cells that show the SP phenotype.

When primary mammosphere derived cells were treated with Rhodamine123, we observed two distinct populations – Rho^high^ and Rho^low^ (Fig. 2Ba). We asked if the Rho^low^ population was the same as ‘SP’. In a simultaneous Rhodamine and Hoechst staining experiment, when SP cells were gated onto the Rhodamine plot, the ‘SP’ (Fig. 2Bb) indeed represented a subpopulation within the Rho^low^ population (Fig. 2Bc).

### Mammosphere forming potential resides within the CD24^low^CD44^high^ subpopulation

A major obstacle in breast stem cell biology is the absence of a specific marker(s) to identify this small population of cells. In some tissues, such as hematopoietic and neuronal tissues, normal and cancer stem cells have been shown to share the same cell surface markers [2], [4]. In the case of breast tissue, breast cancer stem cells have been identified as CD24^low^CD44^high^ cells [3]. We asked if this marker phenotype would also hold true for normal breast stem cells. Flow cytometry analysis of primary mammosphere-derived cells stained for CD24 and CD44 revealed differentially stained populations for the two markers (Fig. 2Ca and Supp Fig. S3A). In order to find out which of these fractions housed cells capable of initiating mammospheres, we sorted out the same number of cells from different quadrants and assessed their sphere forming ability (Fig 2Cb). We observed sphere formation only in the CD24^low^CD44^high^ fraction which represented about 12–15% of the total population, indicating that normal breast stem/progenitor cells can be identified by a CD24^low^CD44^high^ phenotype, in keeping with that for breast cancer stem cells.

In order to understand the correlation between CD24/44 marker profile and SP in mammospheres, we undertook a simultaneous CD 24/44 and SP staining (Fig. 2Da–d). To our surprise we observed that the ‘SP’ cells were present within the CD24^low^CD44^low^ fraction (Fig. 2Dd) that we previously showed fails to initiate mammospheres, suggesting that breast SP cells may lack sphere forming potential. This is in contrast to a previous report which showed that SP from mammospheres had the potential to initiate spheres [19]. However, this previous study had not done a parallel CD24/44 marker staining, and as mentioned earlier, also failed to show a distinct side-population. Based on our data we report that in normal primary mammospheres the SP cells do not fall within the breast cancer stem cell phenotype of CD24^low^CD44^high^.

### Mammospheres fail to form beyond the fifth passage

**Figure 3:**
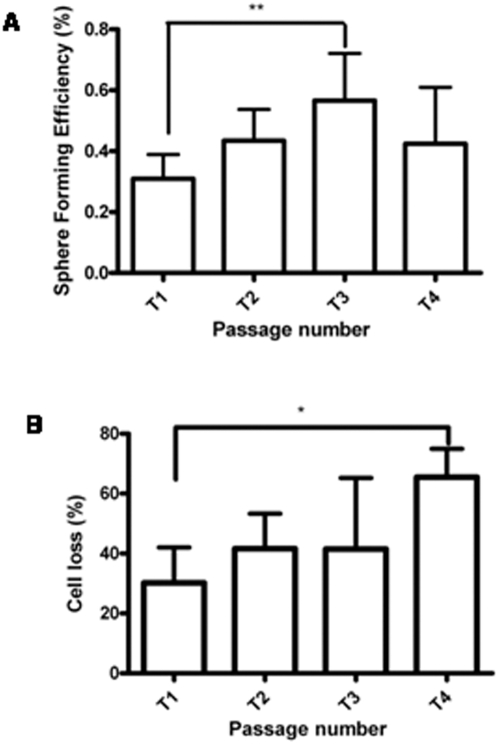
Analysis of serial mammosphere formation. A: Bar graph represents the sphere forming efficiency (SFE) calculated by counting the number of mammospheres formed in a given well using a manually prepared grid and dividing this by the total number of cells seeded in the well, represented as percentage (n = 7). B: Bar graph represents cell death across different passages calculated by taking the difference in live cell count (Trypan blue exclusion) between each passage (n = 5). Statistical analysis for A and B was done using One Way ANOVA; ** signifies p<0.005 . Error bars represent standard deviation.

The ability of primary mammospheres/neurospheres to generate secondary spheres illustrates their self-renewal property [19], [20]. Stem cells within the body, however, need to possess long term self-renewal potential so as to carry out tissue repair and sustain homeostasis throughout adult life. Therefore, to assess the long term self renewal property of human mammary stem cells in vitro, we carried out serial mammosphere assays. We observed an increase in sphere forming efficiency from T1 to T3 (from ∼0.2% to ∼0.6%), but a decline thereafter (Fig. 3A). Interestingly, we failed to detect mammosphere formation beyond the fifth passage in more than 20 independent breast tissues. Trypan blue analysis revealed cell death at every passage with a ten-fold overall cell loss from T1 to T4 (Fig. 3B). In order to confirm that the inability of sphere formation is not due to small number of cells left behind at T4, we used varying cell densities. We observed similar result independent of the starting cell numbers varying from 0.1–1 million cells. After the fifth passage, mostly single cells and few clumps of cells were seen. When left in culture for more than 20–25 days, mostly single cells with few cells containing large vacuoles were detected, but no new mammospheres formed even though trypan blue staining revealed 85% live cells (data not shown). Thus, lack of mammosphere formation could not be attributed to the absence of live cells beyond the fifth passage. Furthermore, the ability of mammospheres to generate acinar structures in matrigel and cells with multilineage differentiation potential under differentiating conditions (Supplementary Fig S4) indicated their stemness to begin with [19]. So, unlike the primary human fetal central nervous system derived neurospheres that could be passaged as spheres up to 30 times [35], adult human breast tissue derived mammospheres could be passaged only five times in culture. Also, unlike the several-fold expansion of neurospheres, mammospheres showed marginal increase in sphere forming efficiency.

### Alterations in proliferation with serial passaging

**Figure 4:**
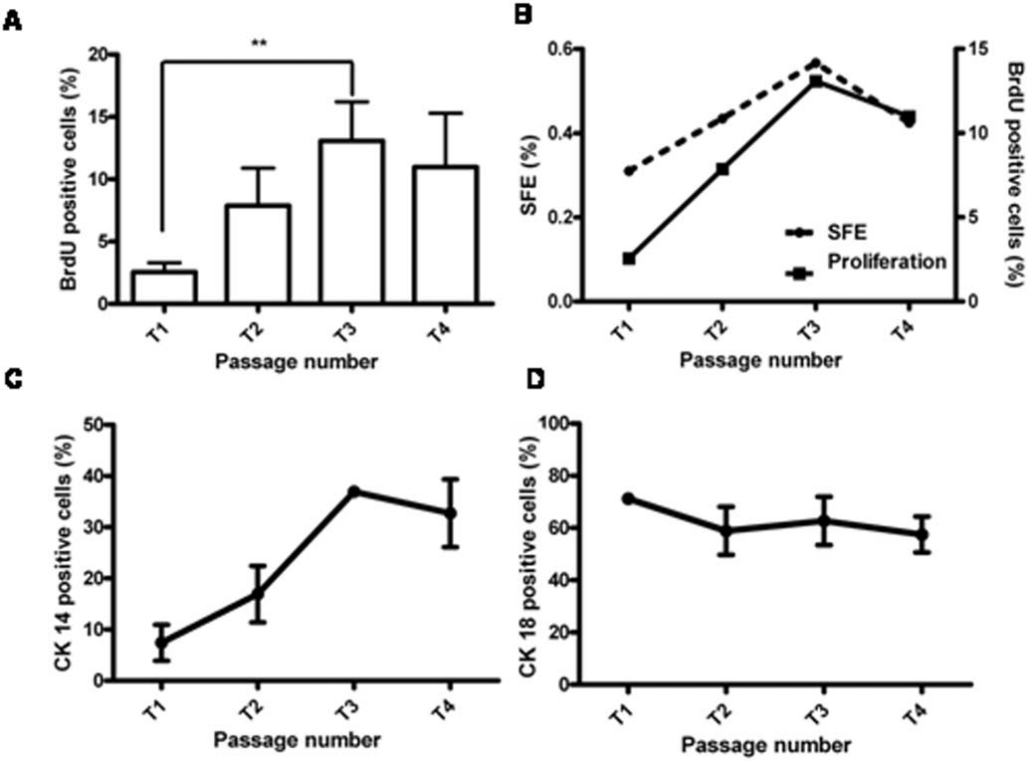
Proliferation and differentiation status of mammosphere derived cells. A: Bar graph represents BrdU incorporation based proliferation analysis at every passage. Ratio of BrdU positive cell and total cell count was taken over random fields and represented as percentage. B: Graph represents comparison of proliferation potential and sphere forming efficiency from T1 to T4 (n = 5). C and D: Graphs represent differentiation status of cells within mammospheres from T1 to T4 passage as assessed by immunostaining for CK14 and CK18. (n = 4). Statistical analysis of this data was done using One Way ANOVA; * signifies p<0.05, ** signifies p<0.005. Error bars represent standard deviation.

Since mammosphere formation involves proliferation, lack of proliferation could also result in the observed lack of mammosphere formation during serial passaging. Accordingly, we tested for the presence of proliferating cells through different passages using BrdU incorporation assay. Mammospheres from T1, T2, T3 and T4 passages contained on an average 3%, 6%, 13% and 10% proliferating cells, based on results obtained from five independent tissues (Fig. 4A). Interestingly, the trend of proliferation of mammosphere derived cells from T1 to T4 corroborated exactly with the trend seen for sphere forming efficiency at each passage (Fig. 4B). However, despite the presence of live, proliferating, and label retaining cells at T4, no new spheres were initiated beyond the 5th passage.

### Increase in the number of differentiated cells with serial passaging

Since only stem/progenitor cells, but not the differentiated cells, have the potential to generate mammospheres, an increase in the number of differentiated cells with serial passaging may also account for reduced mammosphere formation. To determine this, we analyzed the differentiation status based on expression of CK14 and 18 over different passages. The CK14 positive myoepithelial cell population increased from 7–30% from T1–T4, while the CK18 positive luminal epithelial cells population remained uniform, between 60–70%, across passages (Fig. 4C and D). Thus, the increase in the CK14 positive population, suggesting a preferential differentiation towards the myoepithelial lineage in long-term culture of mammospheres, may contribute in part to the reduction of sphere formation with passage, but may not account for the total lack of sphere formation after the fifth passage.

### Mammospheres show an increase in senescent cells with passage

**Figure 5:**
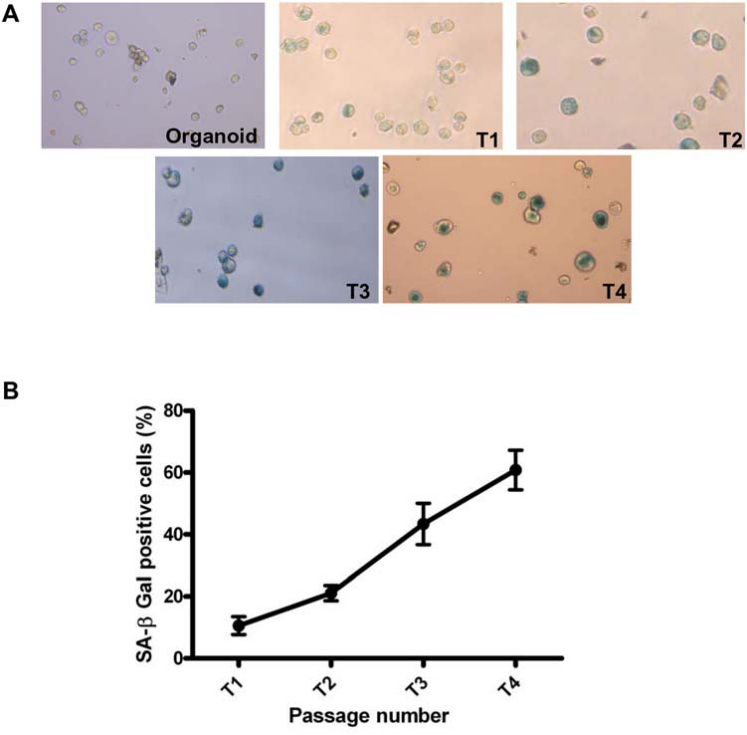
Senescence studies in mammospheres. A: Photomicrographs represent mammosphere-derived cells staining positive (blue color) for senescence associated β Galactosidase (SA β Gal) activity at different passages; ×20 objective. Trypsinized mammospheres were fixed with glutaraldehyde and incubated in X-Gal containing buffer (pH 6.0). B: Graph represents quantitative representation of the senescence profile of mammosphere-derived cells across passages, calculated by dividing the number of β Gal positive cells, as observed under the microscope, by the total number of cells and represented as percentage. (n = 6). Statistical analysis for B was done using One Way ANOVA. Error bars represent standard deviation.

Primary cells in culture are known to undergo senescence after a few passages, which varies from one cell type to the other [36]. In serum containing attachment cultures, primary breast epithelial cells can undergo >20 population doublings before they show signs of senescence [37]. In serum deprived attachment conditions, these cells undergo 15–20 population doublings before hitting senescence [38]. However in serum deprived, suspension cultures, this phenomenon needs to be explored. This led us to test if the cells beyond T5 had attained senescence. Cells at every passage were assayed for senescence associated β Galactosidase (SA- β Gal) activity, a marker of senescent cells [39]. We observed a marked increase in SA-β Gal activity after T2, such that in T4 mammospheres an average of 60% cells were senescent (Fig 5A and B). The cells present in culture beyond the fifth passage, with regular media change every five days, consistently stained positive for SA-β Galactosidase activity. Thus, increase in senescent cells may contribute largely to the inability to generate mammospheres at later passages.

## Discussion

The ability to grow in serum-free and anchorage-independent conditions as multicellular spheroids has been exploited for the isolation of stem/progenitor cells in several systems [19], [20], [40], [41]. Within a given tissue, however, stem cells are embedded in niches which provide them with appropriate cues to divide, differentiate, or remain quiescent. Therefore, in suspension cultures in vitro, in the absence of its natural microenvironment, whether human mammary stem cell properties can be sustained for prolonged periods was not known. We have reported here a detailed characterization of human breast tissue-derived mammospheres over long term culture and demonstrated that mammospheres fail to initiate new spheres beyond the fifth passage.

### Heterogeneity within mammospheres

Morphological characterization of mammospheres indicated that the spheres were of variable sizes ranging between 40 to 110 µm with an increase in smaller size spheres with passage. The difference in the size of the spheres may reflect the cell type of origin of mammospheres, with smaller spheres originating from progenitors, and larger spheres originating from stem cells. Also, the presence of cells of variable sizes within the mammospheres could represent the cellular heterogeneity found within the mammosphere; with smaller cells being the stem cells [42] and the larger cells being the progenitors and differentiated cells.

**Figure 6:**
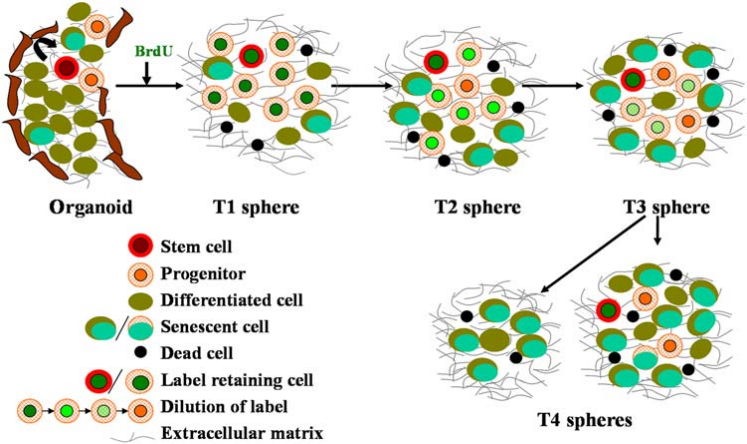
A hypothetical model depicting cellular dynamics within mammospheres at various passages. Based on our observations from serial sphere formation and label retention assays, differentiation and senescence analysis at each passage, we hypothesize that to begin with there is a small number of stem cells, many rapidly dividing progenitors along with a few differentiated cells within each mammosphere. With time, the progenitors differentiate, and the differentiated cells in turn either die or senesce. Some of the progenitors themselves might also senesce. The increasing senescent environment might negatively influence the sphere forming potential of the actual stem cells that may still be present in the senescent milieu at the end of a month, as shown by the label retaining cells.

The label retention experiment (Fig. 1F) further highlighted the existence of a functional hierarchy within mammospheres. Majority of BrdU positive cells in T1 mammospheres may be predominantly progenitors, while the few label retaining cells T4 spheres may represent the true stem cells. Interestingly, some T4 spheres failed to show any label-retaining cells. These spheres were also smaller in size (30–40 µm), further supporting that the smaller spheres may originate from progenitors. By the fifth passage, the vast majority of the progenitor pool would have differentiated (see model Fig. 6), with several of them not even surviving the suspension culture which favors the growth of stem/progenitor cells, thereby accounting for the massive cell loss observed in later passages (Fig. 3B).

### CD24^low^/CD44^high^, and not SP cells, initiate mammospheres

The specific combination of CD24^low^ and CD44^high^ was found to mark breast cancer stem cells [3]. Consistent with this result, our data revealed that in the context of normal breast tissue too, mammosphere forming potential resided only with those exhibiting the CD24^low^CD44^high^ phenotype. Interestingly, only the CD24^low^CD44^high^ sub-fraction from primary mammospheres generated cells of all the other phenotypes (Supp Fig. S3B), thereby indicating that primary mammospheres indeed contain stem cells that can undergo asymmetric division to self-renew and generate the rest of the cells of the mammospheres. Our results however revealed that the mammosphere SP fell within the non-sphere initiating CD24^low^CD44^low^ profile. Even though SP from human breast tissue or mammospheres have so far not been transplanted into humanized, cleared fat pad for evaluation of outgrowths [43], a previous study with murine mammary gland reported that both SP and non-SP population could give mammary outgrowths in vivo [34] thereby indicating that mammary gland stem cells may well be outside the SP regime. Similarly, in murine HSCs, only 10% of the Sca1 (a marker of HSC) positive cells fall in the SP region [31]. Study of SP in murine and human skin also revealed that these cells lacked stem cell characteristics [44]. Similarly, in the neural tissue too, SP cells from embryonic and adult forebrains did not generate NSC-derived colonies, whereas the bulk population did [45]. ‘SP’ fundamentally reflects the status of membrane transporters and thus, may or may not have a strong correlation with long term self renewal potential – the hallmark of stem cells.

### Lack of mammosphere formation beyond the fifth passage

Our results demonstrated a total lack of sphere formation after the fifth passage despite the presence of live (Fig. 3A and B), proliferating (Fig. 4A), and label retaining cells (Fig. 1Fd). We noticed an increased trend towards the myoepithelial lineage with increasing passage, suggesting that manipulation of pathways that regulate the decision between self-renewal and differentiation may help in prolonging mammosphere life-span in culture. Indeed, activation of the Notch and Hedgehog signaling pathway has been shown to increase self-renewal of mammospheres [22], [23]. However, whether they extend mammosphere life-span in culture remains to be determined.

Characterization of the senescence profile of mammospheres at every passage revealed a steady increase in the number of senescent cells from T1 to T4 (Fig. 5A and B). Even though we detected telomerase activity in T1 mammospheres, we failed to both detect hTERT expression (Supp. Fig. S1D) and its activity (Supp. Fig. S1C) in T4 spheres. Owing to the expression of telomerase by adult stem cells, and their ability to grow in anchorage independent conditions – two fundamental properties of cancer cells – it is envisaged that adult stem cells may require fewer genetic alterations to become carcinogenic. Yet, we consistently failed to detect the emergence of any immortalized or transformed cells in our mammosphere cultures spanning over 20 independent breast tissues, indicating that these cells have stringent regulation of cell cycle and DNA damage response.

Senescence is also triggered by non-telomeric signals such as, DNA damage, stress induced, culture conditions, or oncogenic stress [46]. One of the major contributory factors for senescence in the mammospheres culture system could be the culture condition, in particular, oxygen concentration. While the ambient oxygen tension is 20%, the in vivo concentration is between 1–3% in most tissues [47]. The oxygen level is known to be even lower in the stem cell niche in vivo [48], [49]. Reactive oxygen species (ROS) generated by such increased levels of oxygen is known to trigger senescence [46]. To the contrary, culture of cells under low oxygen tension has been shown to prevent or delay their premature senescence [50]–[52]. Thus growing mammospheres in a hypoxic environment may extend its life-span.

So, in the mammosphere culture system, the absence of telomerase in late passage spheres, and continuous exposure to high levels of oxygen, may result in widespread senescence. One possibility is that the stem/progenitor pool itself undergoes senescence. Another possibility is that the presence of a large number of senescent cells around the non-senescent stem/progenitor pool creates an unfavorable ‘niche’ for these primitive cells, thereby altering their self renewal and differentiation potential. The importance of the stem cell niche in regulating stem cell behavior is well recognized [48], [53]. While the cell-cell interactions, cell-extracellular matrix contacts, and response to growth factors may provide a unique niche to the mammary stem cells at early passages, the cues from accumulating senescent cells at later passages may not mimic the same, thereby inhibiting the stem cells from exhibiting their maximum self renewal potential and preventing new sphere formation. This is also in keeping with the growing notion that ‘stemness’ is a state of a cell as a consequence of and maintained by the environment around the cell [54], such that if this environment or ‘niche’ changes, the stemness state of the cell is lost. Thus, in the mammosphere system the label retaining cells within the mammosphere might represent putative stem cells that have lost their stemness in the environment of senescing cells. Thus, either modifications in culture conditions to prevent senescence, or alterations in pathways regulating the decision between self-renewal and differentiation could be exploited to extend mammosphere life-span in culture.

## Supporting Information

Figure S1 Immunostaining and telomerase assay in mammospheres. A and B: Immunostaining of intact T1 mammospheres for CK 14 and 18 followed by optical sectioning of the stained spheres using confocal microscopy. C: TRAP assay done for the detection of functional telomerase in T2 and T4 mammospheres. When viewed on a 12% polyacrylamide gel, while one could observe a typical ladder pattern, which is representative of functionally active telomerase in T2 spheres (Lane 2), no such pattern was seen in T4 spheres (Lane 3). HEK 293T cells were used as positive control (Lane 1) and lysis buffer as negative control (Lane 4). SYBR green was used for detection. D: Detection of telomerase expression from T1 to T4 mammospheres by Real Time PCR. (HeLa cells were used as a positive control; human fibroblasts (hF) and lysis buffer as negative controls).
(1.16 MB DOC)

Figure S2 Single cell sorting of CD24-/lowCD44high cells and SP analysis of mouse bone marrow. Hoechst staining reveals a distinct side population in C56BL/6 mouse bone marrow cells used as positive control for SP staining of mammosphere derived cells. A: Staining of mouse bone marrow cells with Hoechst 33342 shows presence of SP in region R2. B: The disappearance of the side population (R2) in the presence of the transporter blocker Verapamil. C: Single CD24-/lowCD44high sorted single cell derived spheres formed in a 96-well ultra low attachment plate (a, b); Cells derived from the single mammospheres stained for the differentiation markers, CK 14 ( c ) and CK 18 (d).
(1.80 MB DOC)

Figure S3 Analysis of CD24 and CD44 expression. A: Quantitative analysis of expression of the surface markers CD24 and CD44 in three tissues. B: Analysis of expression of CD24 and CD44 by cells sorted from each depicted gates (refer to Fig. 2Cb) after a week in culture in DMEM-F12 containing growth factors seeded in a 24-well Ultra low attachment plate, stained with anti CD24-FITC and anti CD44-PE antibodies. In each profile, the region from which the cells had initially been sorted out has been indicated in the respective region (1–4).
(1.32 MB DOC)

Figure S4 Differentiation potential of mammosphere derived cells. A: Matrigel based 3D differentiation assay, results in formation of spherical (a) and tubular (d) structures within the gel after 15 days in culture. Optical section through the centre of one of the matrigel derived spheres, revealing majority of acinar structures (b and c). These structures stain positive for the differentiation markers, CK 14 (myoepithelial cells) and CK 18 (luminal epithelial cells) (e–f) (blue: Hoechst; green: FITC; scale bar for a and d is 25 µm; for b, c, e and f scale bar is 20 µm). B: Differentiation assay carried out in serum reveals CK 14 positive cells (a), CK 18 positive cells (b) and dual positive cells (c) Arrow indicates cells which are positive for CK 14 but not CK 18 (red: TRITC, green: FITC. Blue: Hoechst; scale bar represents 25 µm)
(2.91 MB TIF)
