# Supplementary figures and images for: Phenotypic and Functional Characterization of Human Mammary Stem/Progenitor Cells in Long Term Culture
Source: PLoS One. 2009 Apr 24;4(4):e5329. doi: 10.1371/journal.pone.0005329 (PMC2669709; doi:10.1371/journal.pone.0005329)

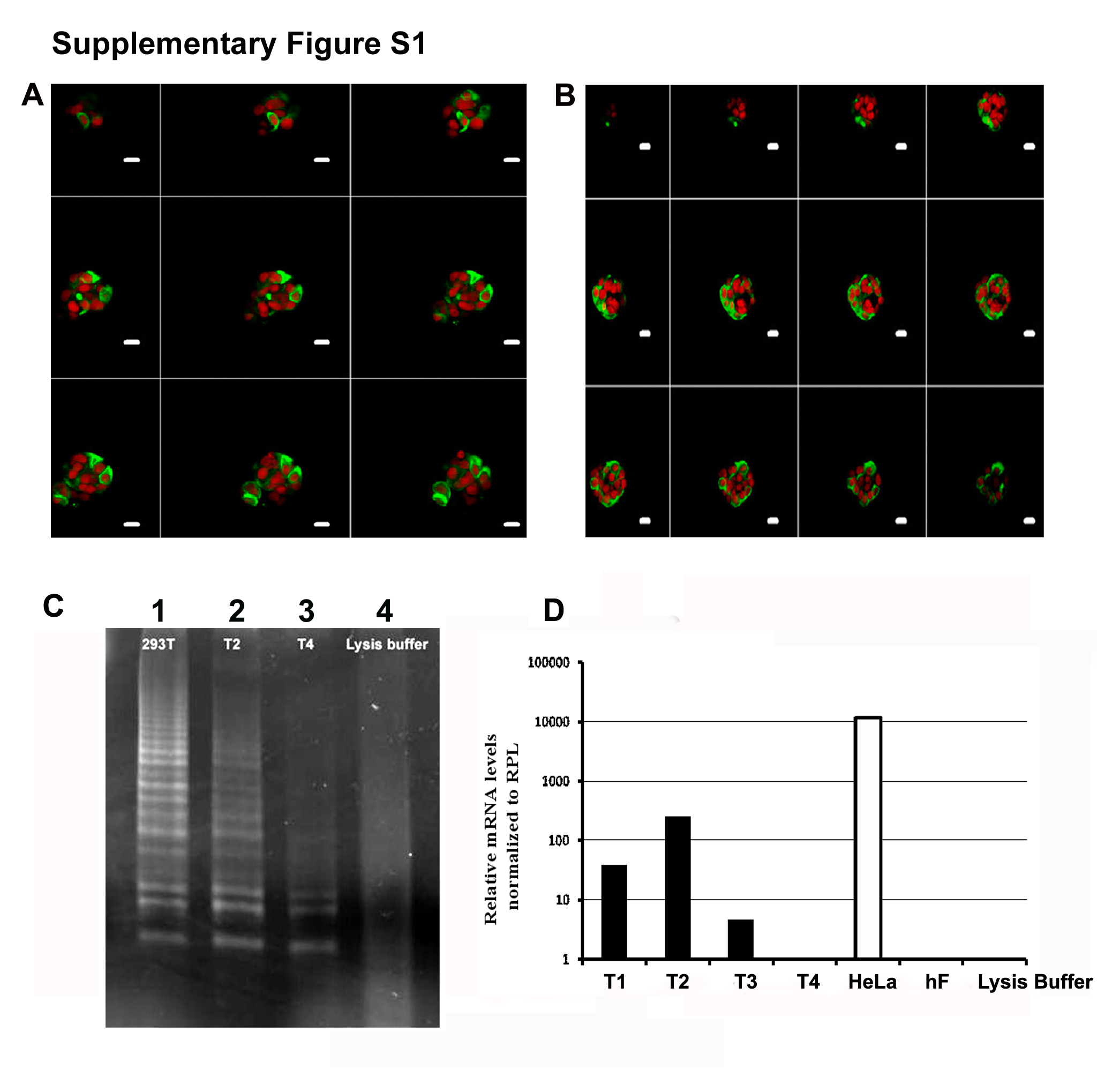

Supplement: Figure S1 — Immunostaining and telomerase assay in mammospheres. A and B: Immunostaining of intact T1 mammospheres for CK 14 and 18 followed by optical sectioning of the stained spheres using confocal microscopy. C: TRAP assay done for the detection of functional telomerase in T2 and T4 mammospheres. When viewed on a 12% polyacrylamide gel, while one could observe a typical ladder pattern, which is representative of functionally active telomerase in T2 spheres (Lane 2), no such pattern was seen in T4 spheres (Lane 3). HEK 293T cells were used as positive control (Lane 1) and lysis buffer as negative control (Lane 4). SYBR green was used for detection. D: Detection of telomerase expression from T1 to T4 mammospheres by Real Time PCR. (HeLa cells were used as a positive control; human fibroblasts (hF) and lysis buffer as negative controls). (1.16 MB DOC) [file pone.0005329.s001.doc]

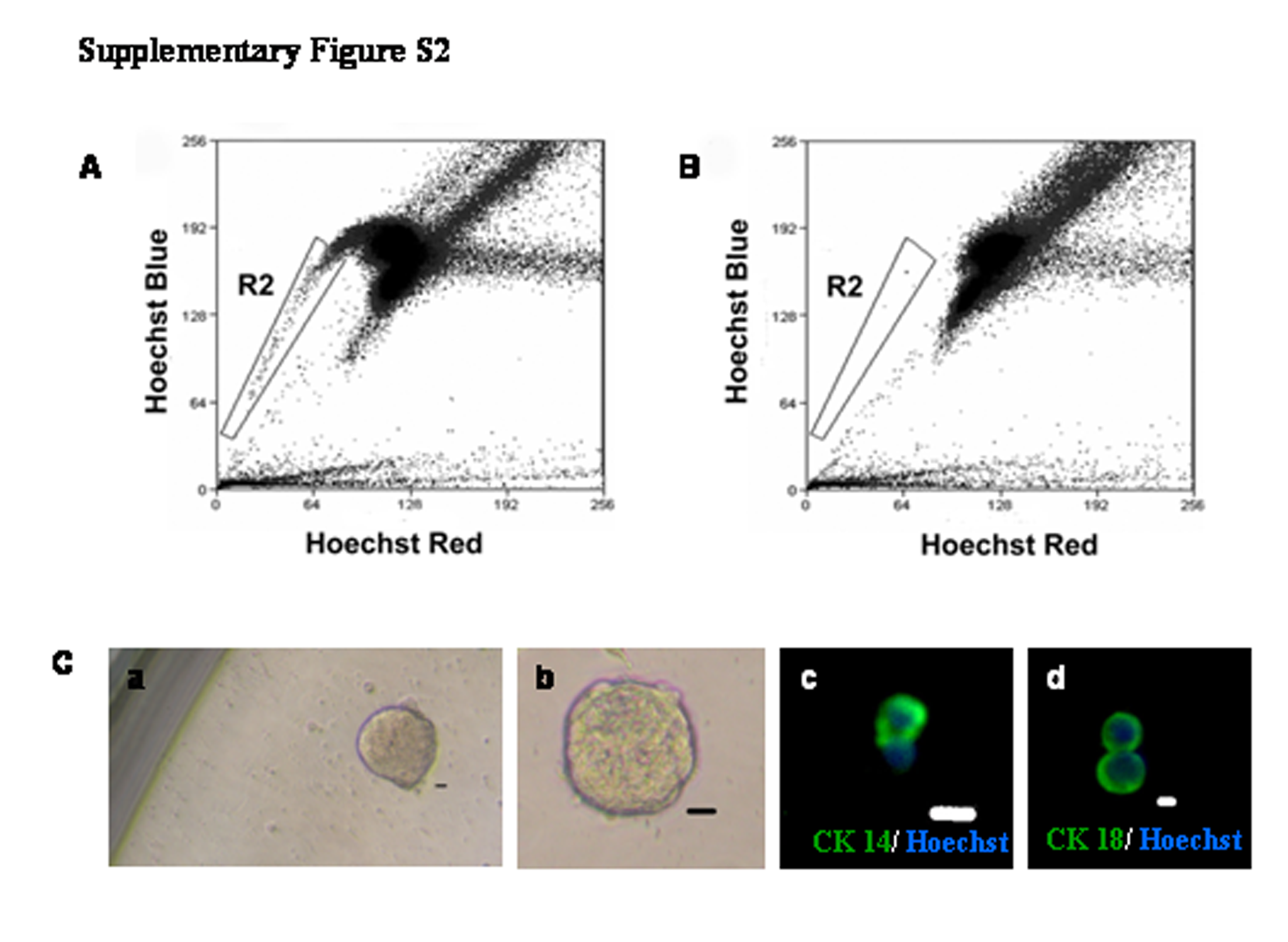

Supplement: Figure S2 — Single cell sorting of CD24-/lowCD44high cells and SP analysis of mouse bone marrow. Hoechst staining reveals a distinct side population in C56BL/6 mouse bone marrow cells used as positive control for SP staining of mammosphere derived cells. A: Staining of mouse bone marrow cells with Hoechst 33342 shows presence of SP in region R2. B: The disappearance of the side population (R2) in the presence of the transporter blocker Verapamil. C: Single CD24-/lowCD44high sorted single cell derived spheres formed in a 96-well ultra low attachment plate (a, b); Cells derived from the single mammospheres stained for the differentiation markers, CK 14 ( c ) and CK 18 (d). (1.80 MB DOC) [file pone.0005329.s002.doc]

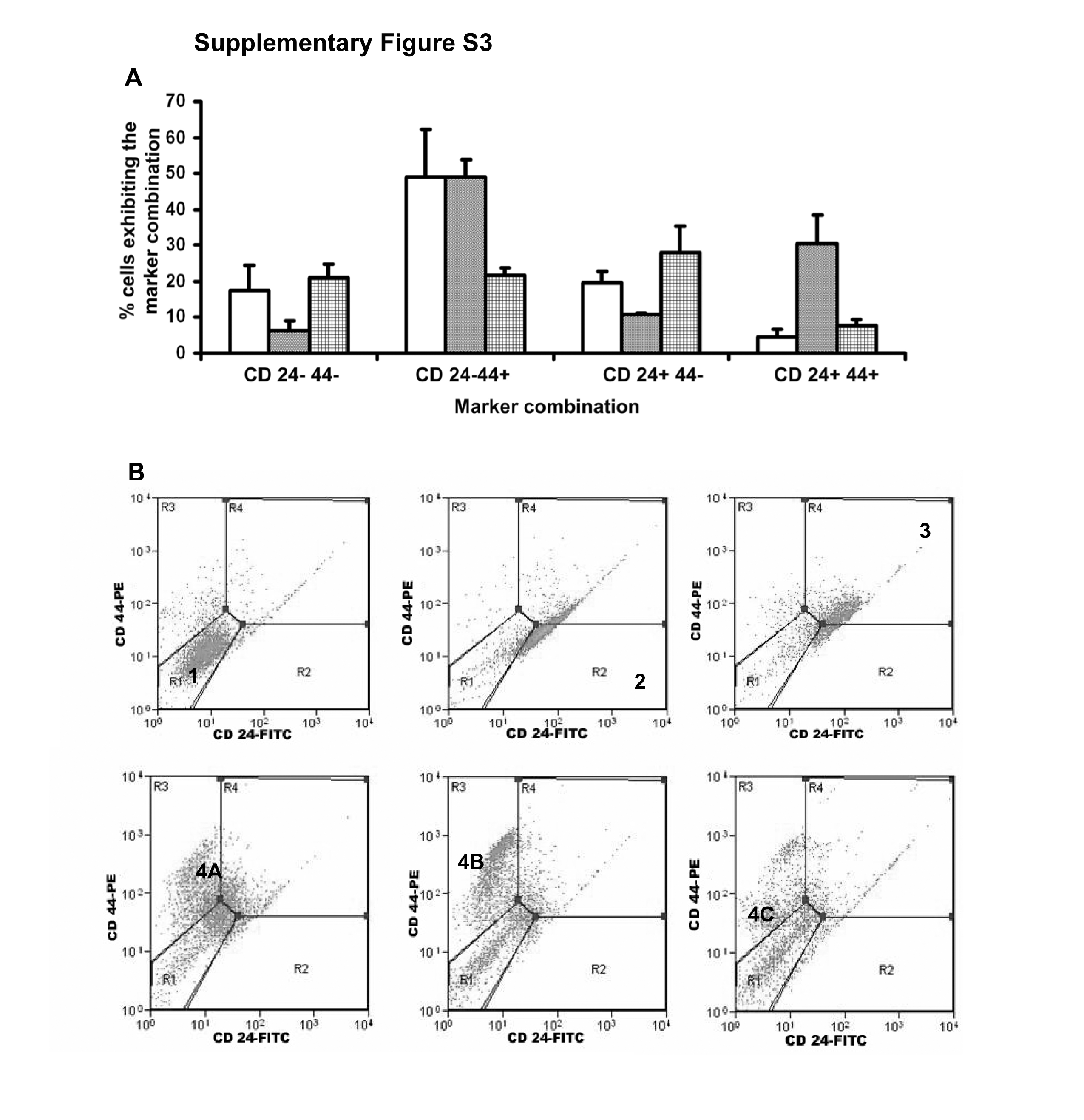

Supplement: Figure S3 — Analysis of CD24 and CD44 expression. A: Quantitative analysis of expression of the surface markers CD24 and CD44 in three tissues. B: Analysis of expression of CD24 and CD44 by cells sorted from each depicted gates (refer to Fig. 2Cb) after a week in culture in DMEM-F12 containing growth factors seeded in a 24-well Ultra low attachment plate, stained with anti CD24-FITC and anti CD44-PE antibodies. In each profile, the region from which the cells had initially been sorted out has been indicated in the respective region (1–4). (1.32 MB DOC) [file pone.0005329.s003.doc]

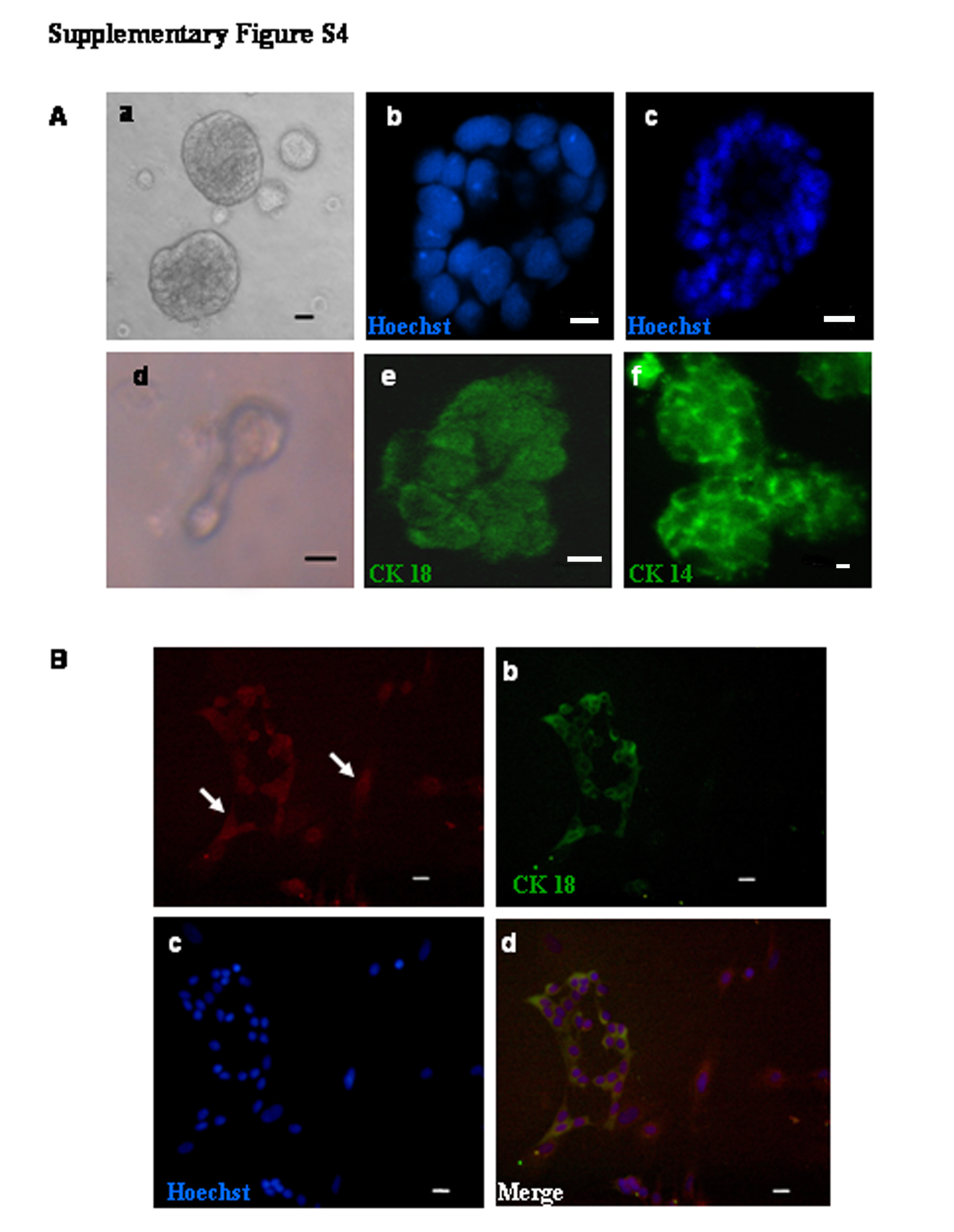

Supplement: Figure S4 — Differentiation potential of mammosphere derived cells. A: Matrigel based 3D differentiation assay, results in formation of spherical (a) and tubular (d) structures within the gel after 15 days in culture. Optical section through the centre of one of the matrigel derived spheres, revealing majority of acinar structures (b and c). These structures stain positive for the differentiation markers, CK 14 (myoepithelial cells) and CK 18 (luminal epithelial cells) (e–f) (blue: Hoechst; green: FITC; scale bar for a and d is 25 µm; for b, c, e and f scale bar is 20 µm). B: Differentiation assay carried out in serum reveals CK 14 positive cells (a), CK 18 positive cells (b) and dual positive cells (c) Arrow indicates cells which are positive for CK 14 but not CK 18 (red: TRITC, green: FITC. Blue: Hoechst; scale bar represents 25 µm) (2.91 MB TIF) [file pone.0005329.s004.tif]
